# A Transcriptomic Analysis of Higher-Order Ecological Interactions in a Eukaryotic Model Microbial Ecosystem

**DOI:** 10.1128/msphere.00436-22

**Published:** 2022-10-19

**Authors:** C. G. Conacher, R. K. Naidoo-Blassoples, D. Rossouw, F. F. Bauer

**Affiliations:** a South African Grape and Wine Research Institute, Department of Viticulture and Oenology, Stellenbosch University, Stellenbosch, South Africa; DOE Joint Genome Institute

**Keywords:** yeast-yeast interactions, higher-order interactions, yeast multispecies ecosystems, ecoevolution, fermentation bioprocesses, RNA-Seq, wine, microbial consortia, microbial ecology, microbial interactions, wine ecosystem, yeast interactions

## Abstract

Nonlinear ecological interactions within microbial ecosystems and their contribution to ecosystem functioning remain largely unexplored. Higher-order interactions, or interactions in systems comprised of more than two members that cannot be explained by cumulative pairwise interactions, are particularly understudied, especially in eukaryotic microorganisms. The wine fermentation ecosystem presents an ideal model to study yeast ecosystem establishment and functioning. Some pairwise ecological interactions between wine yeast species have been characterized, but very little is known about how more complex, multispecies systems function. Here, we evaluated nonlinear ecosystem properties by determining the transcriptomic response of Saccharomyces cerevisiae to pairwise versus tri-species culture. The transcriptome revealed that genes expressed during pairwise coculture were enriched in the tri-species data set but also that just under half of the data set comprised unique genes attributed to a higher-order response. Through interactive protein-association network visualizations, a holistic cell-wide view of the gene expression data was generated, which highlighted known stress response and metabolic adaptation mechanisms which were specifically activated during tri-species growth. Further, extracellular metabolite data corroborated that the observed differences were a result of a biotic stress response. This provides exciting new evidence showing the presence of higher-order interactions within a model microbial ecosystem.

**IMPORTANCE** Higher-order interactions are one of the major blind spots in our understanding of microbial ecosystems. These systems remain largely unpredictable and are characterized by nonlinear dynamics, in particular when the system is comprised of more than two entities. By evaluating the transcriptomic response of S. cerevisiae to an increase in culture complexity from a single species to two- and three-species systems, we were able to confirm the presence of a unique response in the more complex setting that could not be explained by the responses observed at the pairwise level. This is the first data set that provides molecular targets for further analysis to explain unpredictable ecosystem dynamics in yeast.

## INTRODUCTION

Microbial communities are essential service providers to humans, performing functions ranging from digestion to bioremediation. Microbial ecosystems are genetically and functionally diverse, allowing them to perform a myriad of bioprocesses with resilience and a capacity to dynamically respond to fluctuations in their environment (1, 2). Historically, humans have primarily exploited the functionality of single microorganisms, for example, Saccharomyces cerevisiae, for biotechnological applications, such as heterologous enzyme production and bioethanol production, as well as food and beverage production. However, the use of monocultures in biotechnological processes has reached somewhat of an innovation ceiling in more complex bioremediation and fermentative bioprocesses, where emphasis is shifting from modifying a single strain to perform many functions to trying to exploit complementary properties of multiple strains and species within a custom-designed microbial ecosystem (3, 4).

There are, however, significant challenges that limit our ability to harness microbial ecosystems. One of these is the fact that there is a lack of predictive understanding of the mechanisms that govern the establishment and functioning of these ecosystems (5). In terms of the molecular mechanisms that govern yeast-yeast interactions, the current state of knowledge is largely based on binary, i.e., two-species, systems (6–13). These are comparatively easier to investigate than multispecies systems, given their better predictability, and such simpler systems can provide foundationally important data sets before attempting to unravel more complex systems. These studies have investigated the responses of yeast species to each other at the transcriptomic and proteomic level and have focused on the response of S. cerevisiae to other yeast species. A few studies have also reported on non-*Saccharomyces* partner responses, including Torulaspora delbrueckii and Lachancea thermotolerans, which are both popular choices for industrial fermentations given their strong fermentative capacity and contribution to positive sensory qualities (7–9). The focus on S. cerevisiae in most of these studies is linked to this species playing a dominant role in the wine ecosystem while also being a model organism with an excellent molecular toolbox and research archive from which to draw upon. The conclusions of these studies have shown that there are significant impacts on S. cerevisiae at the transcriptional and translational level in response to mixed-species culture, and more interestingly, there are indeed species-specific impacts on S. cerevisiae as well.

In contrast, very little is known about the influence of nonbinary interactions within yeast ecosystems, and this remains a major research challenge within the broader field of microbial ecology (3, 5, 14). Higher-order interactions are nonlinear effects on the existent interactions (and functioning) of a microbial community, which happen when either pairwise interactions are perturbed by the presence of other interactors or completely new properties emerge as a result of a specific combination of microbial role players (15). Currently, the available quantitative data of higher-order interactions in microbial ecology are dominated by bacterial communities only (3, 4, 14, 16–20). In yeast, far less is known about higher-order interactions, with the best available data being population dynamics that have been collected during fermentations with inoculated yeast consortia. These are limited in terms of comparing population dynamics in cultures of increasing complexity, so the emergence of any higher-order effects is masked (21–27).

Here, we have sought to study the emergence of higher-order interactions at the transcriptomic level in S. cerevisiae within the simplest possible consortium of three wine-associated yeast species. The consortium was comprised of *Lachancea thermotolerans*, Torulaspora delbrueckii, and S. cerevisiae, three species that are present in significant cellular concentrations in natural wine fermentations globally and that can be considered core elements of the evolutionarily relevant wine microbial ecosystem (28). Population dynamics, major metabolite concentrations, and the mRNA transcriptome of S. cerevisiae were compared between mono-, bi-, and tri-species culture. We report on the species-specific impacts of the pairwise cocultures on S. cerevisiae, contributing to our understanding of the ecological interactions at play and allowing for comparison with previous pairwise studies that investigated similar species. By eliminating the signature of pairwise interactions from the consortium data set, we were able to reveal the presence of a response unique to consortium growth, which alludes to a possible higher-order impact on S. cerevisiae in response to more than one interacting species. These data contribute to our broader understanding of yeast-yeast interactions within the wine fermentation ecosystem and, importantly, give a first insight into the potential mechanisms that allow S. cerevisiae to consistently dominate this ecosystem. These findings have implications for the future design of synthetic yeast ecosystems, as well as our fundamental understanding of the role of biotic stress on the establishment and functioning of such ecosystems.

## RESULTS

### Population dynamics of the synthetic yeast consortium.

**FIG 1:**
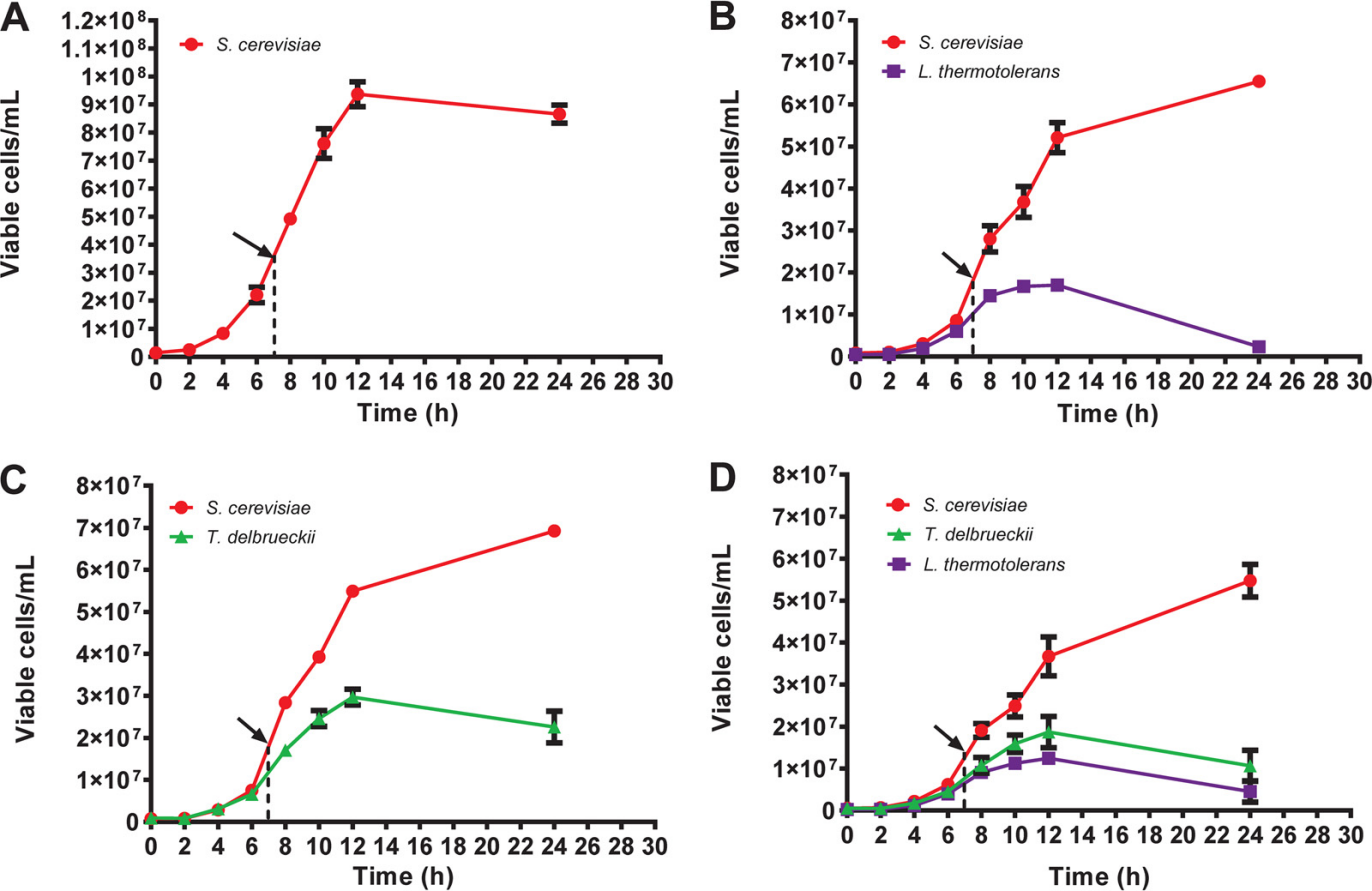
Population dynamics of mono-, bi-, and tri-species cultures, grown at 30°C in optimized YNB growth medium with aeration and agitation. Black arrows indicate sampling point for RNA sequencing. Red circles, Saccharomyces cerevisiae; purple squares, *Lachancea thermotolerans*; green triangles, Torulaspora delbrueckii.

The growth levels of S. cerevisiae were compared between monoculture, pairwise, and three-species culture settings (Fig. 1). S. cerevisiae was the dominant species in each mixed-culture scenario (25, 26, 29–32). In the pairwise cocultures with S. cerevisiae, *L. thermotolerans* growth appeared to be more severely attenuated than that of *T. delbrueckii* (Fig. 1B and C). Cell numbers of S. cerevisiae throughout growth in both of these pairings were nearly identical. S. cerevisiae achieved dominance after 8 h of coculture in both pairwise experiments. These pairwise population dynamics were also reflected in the three-way population dynamics, with S. cerevisiae being the dominant species and *T. delbrueckii* having slightly higher cell numbers than *L. thermotolerans* from 8 h onward (Fig. 1D). The growth patterns of S. cerevisiae in pairwise (Fig. 1B and C) and consortium (Fig. 1D) cultures were highly similar. One significant difference between monoculture and cocultures was that the monoculture reached stationary phase after 12 h of growth (Fig. 1A) while mixed cultures showed an extended growth phase of S. cerevisiae after 12 h (Fig. 1B to D). Overall, these cell number-based trends indicate that in terms of relative abundances, the pairwise population dynamics were a good predictor for the dynamics in the more complex tri-species system.

### Differential expression analysis of S. cerevisiae in mixed-species cultures.

**FIG 2:**
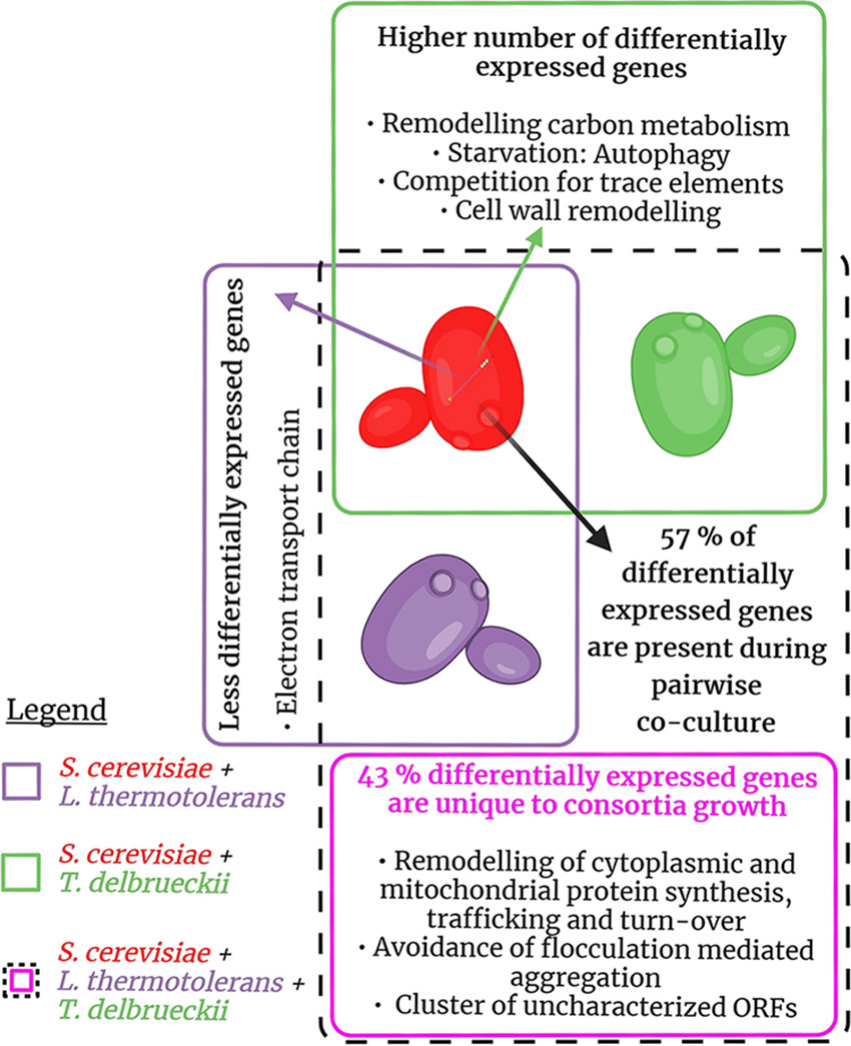
Summary of the transcriptomic response of S. cerevisiae to pairwise and three-species consortium growth. ORFs, open reading frames.

To characterize gene expression programs of S. cerevisiae associated with potential higher-order interactions within a three-species yeast consortium, differential expression levels of genes were compared between the consortium and every possible pairwise combination within the consortium (Fig. 2). To contextualize these results, it is important to consider the state of the ecosystem at the sampling point (and therefore the selected experimental settings and sample point selection), especially given the fact that only one time point is being evaluated. After 7 h of growth, in all mixed-culture settings, S. cerevisiae has just begun to dominate competing species in terms of cell numbers and is in the early exponential growth phase. This time point represents an important point in the growth of S. cerevisiae, where its dominant attributes are emerging at the phenotypic level while it still has similar cell numbers as the other species. At this point, the total biomass and the sugar consumption rate, and therefore total metabolic activity, in each sample can be considered similar.

**FIG 3:**
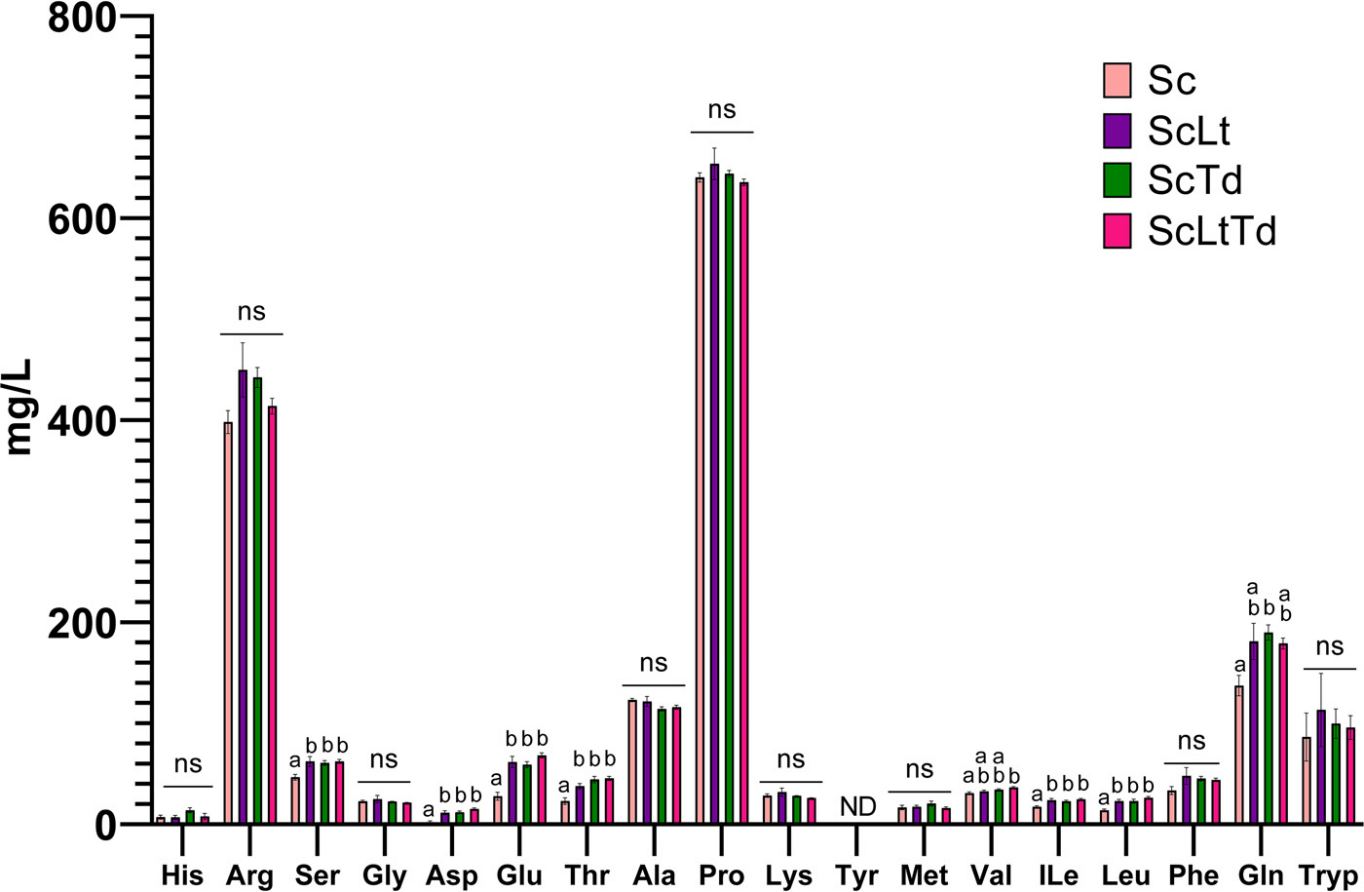
Measured absolute amino acid concentrations in the supernatants of S. cerevisiae monoculture and mixed-species cultures. Values are represented as the mean from four biological repeats. Statistically significant differences in amino acid concentrations between different cultures are labeled with differing letters and were calculated by ANOVA followed by Tukey’s multiple-comparison test. Sc, Saccharomyces cerevisiae; Lt, *Lachancea thermotolerans*; Td, Torulaspora delbrueckii; ns, not significant.

Given the batch culture conditions, it was also essential to keep the nutritional state of the growth medium as consistent as possible between samples. The sugar (glucose and fructose), glycerol, yeast assimilable nitrogen (YAN), organic acid (citric acid, tartaric acid, malic acid, succinic acid, lactic acid, and acetic acid), and methanol concentrations of the cultures were all similar (see Table S2.1 in the supplemental material; analysis of variance [ANOVA], *P* ≫ 0.05), which supports the fact that all the cultures were at similar points of growth, thereby reducing the likelihood that the differences observed here were due to various physiological states linked to nutrient availability or growth phases. Overall, ethanol concentrations were similarly low, as expected at early time points, with slightly lower ethanol concentrations in the coculture samples (average across all mixed cultures: 0.47% [vol/vol]) than in the monoculture (0.60% [vol/vol]) (Table S2.1). There were also differences in amino acid concentrations, specifically serine, aspartic acid, glutamic acid, threonine, valine, isoleucine, leucine, and glutamine (Fig. 3 and Table S2.2). This is in line with known strain-specific preferential uptake of amino acids which has previously been observed in monoculture and mixed-culture contexts (33–35). The causation behind these differences in absolute concentrations of amino acids cannot be determined here since it is not possible with the current methodology to follow uptake and/or release of amino acids by a particular species during mixed culture. Still, the fact remains that there are different available absolute concentrations of particular amino acids in the extracellular environment of these yeast cells, and this may indeed be a highly relevant factor of yeast-yeast interaction to consider.

10.1128/msphere.00436-22.9 TABLE S2 (1) Metabolite concentrations of the supernatant collected from four biological repeat samples used for RNA Sequencing. (2) Amino acid concentrations of the supernatant collected from four biological repeat samples used for RNA Sequencing. Download Table S2, DOCX file, 0.02 MB. Copyright © 2022 Conacher et al. 2022 Conacher et al. https://creativecommons.org/licenses/by/4.0/ This content is distributed under the terms of the Creative Commons Attribution 4.0 International license.

**FIG 4:**
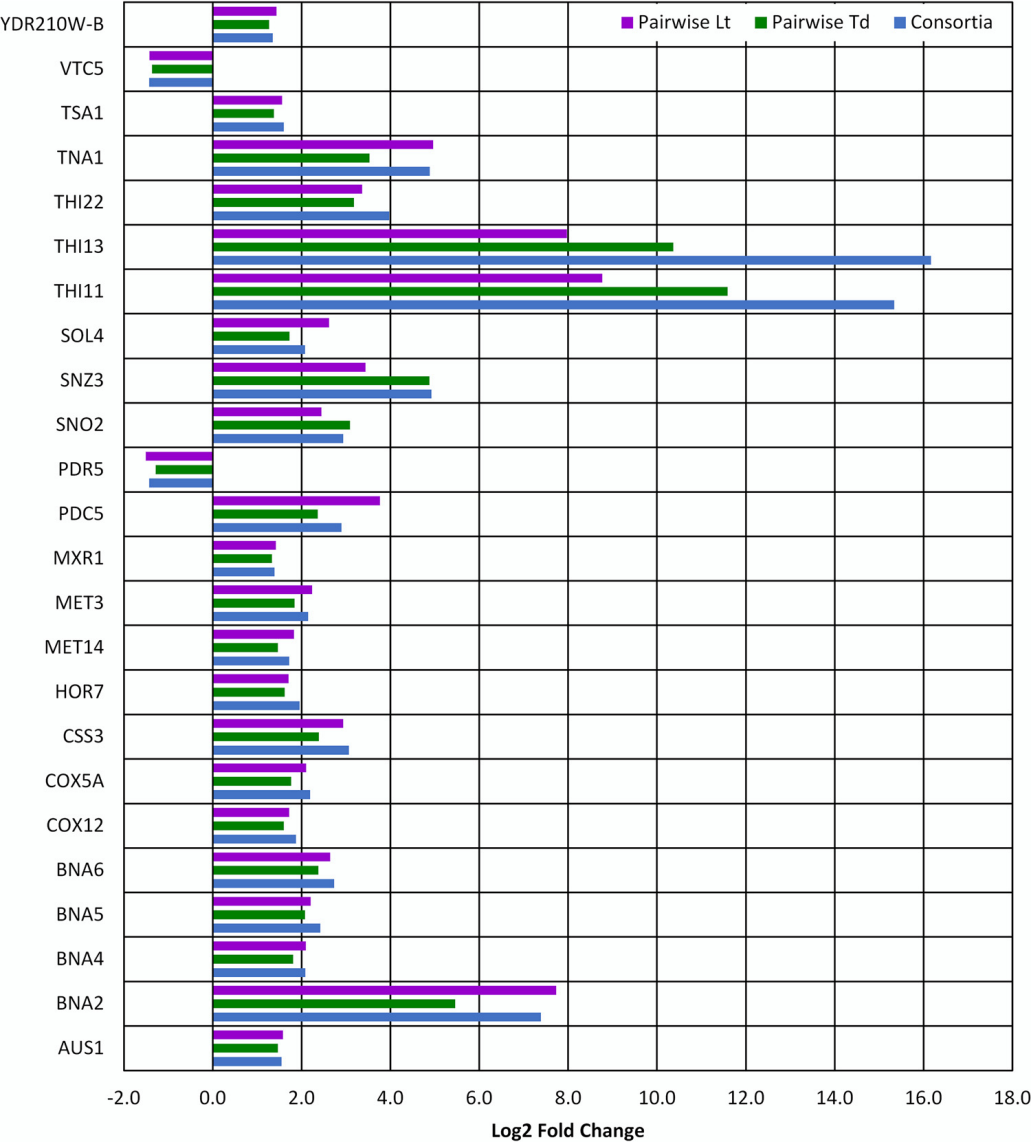
Differentially expressed genes in S. cerevisiae that were present in both pairwise cocultures (with *L*. *thermotolerans* and *T*. *delbrueckii*, respectively) as well as in consortium culture with all three yeast species.

**(i) Generalized response of S. cerevisiae to mixed-species culture.** In all tested species combinations, 24 genes were consistently differentially expressed (Fig. 4). These genes were similarly up- or downregulated under all conditions, and the differential expression levels were highly correlated between all conditions. Notably, these commonly affected genes were also some of the most highly upregulated and statistically significant (Data Set S1) under each tested condition.

10.1128/msphere.00436-22.10 DATA SET S1 RNA-Seq data sets. Filtered differentially expressed gene lists under coculture conditions, preprocessing analysis comparison between 8- and 12-bp cutoff conditions, filtering of consortium DEG list, and functional enrichment analysis summary across all coculture conditions. Download Data Set S1, XLSX file, 1.0 MB. Copyright © 2022 Conacher et al. 2022 Conacher et al. https://creativecommons.org/licenses/by/4.0/ This content is distributed under the terms of the Creative Commons Attribution 4.0 International license.

Two major functional pathways were upregulated within this group, namely, thiamine biosynthesis (THI11, THI13, THI22, SNZ3, SNO2) and NAD^+^ biosynthesis (*de novo* pathway: BNA2, BNA4, BNA5, BNA6; salvage pathway: TNA1). Furthermore, a key stress-protective glycerol synthesis gene, GPD1 (HOR7), oxidative stress-associated genes (TSA1, MXR1), and a DNA replication stress gene (SOL4) were all upregulated, providing evidence for a potential link to the stress-protective need for thiamine.

**FIG 5:**
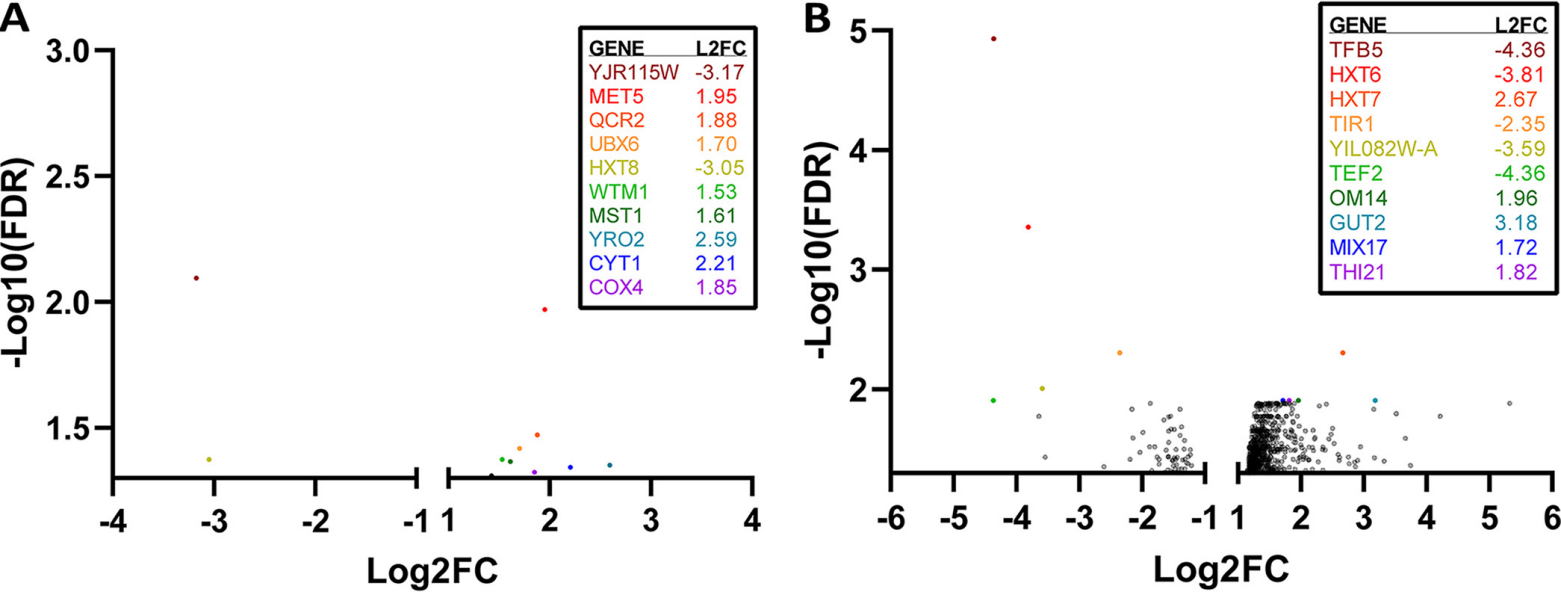
Volcano plots of differentially expressed genes in S. cerevisiae in response to coculture with *L. thermotolerans* (A) or *T. delbrueckii* (B), excluding the genes reported in Fig. 4. Minimum −log_10_(FDR) of 1.3 and fold change (FC) cutoff of 1 and −1. Genes with the top 10 highest statistical significances are shown and color coded to the graph.

**(ii) S. cerevisiae shows species-specific transcriptome remodeling during pairwise culture.** Differentially expressed genes (DEGs) unique to each pairing with either *L. thermotolerans* or *T. delbrueckii* were then comparatively assessed. There was a remarkable difference in the number of affected genes between the two pairings, with *T. delbrueckii* (807 DEGs) eliciting over 20-fold more of a response than *L. thermotolerans* (35 DEGs) (Fig. 5).

Pairwise interaction with *L. thermotolerans* induced upregulation of a few genes involved in the respiratory electron transport chain, specifically mitochondrial ATP synthesis (COX4, CYT1, QCR2) and ubiquinone biosynthesis (COQ6) genes (Fig. S3). Further, the lesser-studied putative hexose transporter (HXT8) was downregulated. Taken with the upregulation of pyruvate decarboxylase gene PDC5 (expressed under all conditions), this suggests a shift to simultaneous fermentative and respiratory metabolism, common to Crabtree-positive yeasts under high-glucose, aerobic conditions. Other upregulated genes included a ubiquitin biosynthesis gene (UBX6), a weak acid stress response gene (YRO1), and a transcriptional modulator of meiosis, gene silencing, and stress-induced RNR genes (WTM1), as well as amino acid biosynthesis genes (MET5, MST1). Interestingly, the gene with the highest statistical significance value (excluding those genes mentioned in Fig. 4) was an uncharacterized gene (YJR115W), which was downregulated, similarly to a previous study that evaluated S. cerevisiae and *L. thermotolerans* pairings (7), highlighting this gene as a potential target for future functional annotation studies.

10.1128/msphere.00436-22.4 FIG S3 Differentially expressed genes in S. cerevisiae in response to coculture with *L. thermotolerans*, excluding the genes reported in Fig. S2. The nodes are colored according to log_2_ fold change values and sized according to statistical significance values. Image and network created in Cytoscape. Figure is for illustrative purposes only; please see interactive network files. Download FIG S3, PDF file, 0.01 MB. Copyright © 2022 Conacher et al. 2022 Conacher et al. https://creativecommons.org/licenses/by/4.0/ This content is distributed under the terms of the Creative Commons Attribution 4.0 International license.

10.1128/msphere.00436-22.3 FIG S2 Differentially expressed genes in S. cerevisiae that were present in both pairwise cocultures as well as consortium culture. The nodes are colored according to log_2_ fold change values, expressed as an average value between all tested conditions. Image and network created in Cytoscape. Figure is for illustrative purposes only; please see interactive network files. Download FIG S2, PDF file, 0.03 MB. Copyright © 2022 Conacher et al. 2022 Conacher et al. https://creativecommons.org/licenses/by/4.0/ This content is distributed under the terms of the Creative Commons Attribution 4.0 International license.

*T. delbrueckii* stimulated a more extensive metabolic shift in S. cerevisiae (Fig. 5). There was major remodeling of central carbon metabolism, with activation of glucose metabolism, which may be a competitive response to increase uptake of this preferred carbon source (Fig. S4, clusters 1, 3, and 6). Glucose sensing and carbon catabolite repression (CCR) regulators were differentially expressed, including two target hexose transporters (HXT6, HXT7) involved in CCR that had high statistical significance within the data set (Fig. 5 and Fig. S4, cluster 6). There appeared to also be simultaneous fermentative (Fig. S4, cluster 10) and respiratory (Fig. S4, cluster 3) metabolism, albeit with more pronounced impacts on respiration. Specifically, there was upregulation of respiratory genes involved in the mitochondrial electron transport chain (Fig. S4, cluster 3), reorganization of mitochondrial structure (Fig. S4, clusters 18, 24, and 25), increased flux through the tricarboxylic acid (TCA) cycle (Fig. S4, cluster 3), and activation of both the oxidative and nonoxidative branches of the pentose-phosphate pathway (Fig. S4, cluster 1). Further, the SIP2 gene, central to the glucose starvation response, was also upregulated. Interestingly, all genes required for trehalose biosynthesis and regulation (Fig. S4, cluster 1) were upregulated, as well as the two genes involved in trehalose-to-glucose catabolism (NTH1, ATH1; cluster 1), indicating that S. cerevisiae may be preparing to store excess glucose and recycle it as a means of competition. Trehalose is also known to be involved in a number of cellular stress responses (36, 37).

10.1128/msphere.00436-22.5 FIG S4 Differentially expressed genes in S. cerevisiae in response to coculture with *T. delbrueckii*, excluding the genes reported in Fig. S2 and outlier YLR162W-A. Nodes are colored according to log_2_ fold change values and sized according to statistical significance values. Image and network created in Cytoscape. Figure is for illustrative purposes only; please see interactive network files. Download FIG S4, PDF file, 0.7 MB. Copyright © 2022 Conacher et al. 2022 Conacher et al. https://creativecommons.org/licenses/by/4.0/ This content is distributed under the terms of the Creative Commons Attribution 4.0 International license.

Consistent with a response to starvation, several autophagy and autophagy-associated genes were differentially expressed. Autophagy is induced during nutrient starvation and is the process of the cell cannibalizing organelles and using the resultant by-products to maintain metabolic homeostasis (38, 39). There were signs of activation of signaling cascades mediated by Ser/Thr protein phosphatases (Fig. S4, clusters 5 and 39), which are essential in nutrient sensing, and upregulation of Ras-like protein 2 (RAS2), which is involved in responding to nitrogen starvation (Fig. S4, cluster 44). Macroautophagy genes were upregulated (Fig. S4, clusters 8, 30, and 40), as were associated intracellular vesicular trafficking and secretion genes including endocytic genes (Fig. S4, cluster 4), soluble *N*-ethylmaleimide-sensitive factor attachment protein receptor (SNARE) complex-associated genes (Fig. S4, cluster 20), and endoplasmic reticulum (ER)-associated secretory genes (Fig. S4, cluster 29). Impacts on transcription were also present, with differential expression of genes involved in transcription by RNA polymerase II (Fig. S4, cluster 37), upregulation of RNA helicases (Fig. S4, cluster 14), and upregulation of transcription activators (Fig. S4, cluster 42). The second largest network cluster, cluster 2 (Fig. S4), illustrated a shift in protein turnover and stress-related changes in translational programs. Genes involved in cytoplasmic translation were downregulated, indicating a cessation of cytoplasmic translation, while genes involved in proteolysis and protein ubiquitination were upregulated, which is consistent with autophagy-related protein catabolism and recycling. There was upregulation of genes involved in protein misfolding, including those involved in endoplasmic reticulum (ER)-associated protein degradation (Fig. S4, cluster 27). Further, lipid droplet catabolism, which is also a central autophagic mechanism, was upregulated (Fig. S4, clusters 16, 22, 35, and 38) (39).

In terms of major carbon and nitrogen metabolism, there appeared to be competition for glucose, as reported above, and there appears to be remodeling in response to available nitrogen sources and a need for sulfur-containing amino acids. Specifically, a general amino acid permease was upregulated (AGP2, cluster 13, Fig. S4), signaling a lack of preferred nitrogen sources, and the uptake of sulfate and biosynthesis of sulfur-containing amino acids, particularly methionine, were upregulated (Fig. S4, clusters 12 and 15). Interestingly, the metabolite data largely do not reflect starvation conditions in terms of available nitrogen and carbon; however, there are indeed differences in the amino acid concentrations tested here, and preferred amino acid concentrations may have contributed to stimulating this response. Further, vitamins and trace elements may have been limiting, since gene targets related to thiamine (Fig. S4, cluster 9) and zinc (Fig. S4, cluster 23), as well as copper and iron (Fig. S4, cluster 17), were differentially expressed.

General oxidative (Fig. S4, cluster 31) and osmotic (Fig. S4, cluster 11) stress response genes were upregulated, as well as peroxisome biogenesis genes (Fig. S4, cluster 41), which are involved in oxidative stress management (40, 41). In addition, genes involved in DNA repair were also upregulated, indicating some DNA replication stress (Fig. S4, cluster 43).

Finally, in agreement with the majority of previous coculture analyses, there were significant alterations in the expression of cell envelope-associated genes (8, 9, 13). Cell wall organization and biogenesis genes were upregulated (Fig. S4, clusters 7, 26, and 33), and cell wall mannoproteins were also impacted, with TIR1 having high statistical significance within this group of genes (Fig. 5 and Fig. S4, cluster 33). Components of eisosomes, which are distinct, dynamic plasma membrane subdomains which have been shown to play a role in responding to membrane stressors, were also upregulated (Fig. S4, cluster 11) (42).

The overall response showed similarities to the environmental stress response (ESR) program, a generalized response to varied cellular stresses, which has previously been observed in other S. cerevisiae and *T. delbrueckii* coculture studies (8, 9, 13, 43), with notable parallels to starvation responses, autophagy in particular.

**FIG 6:**
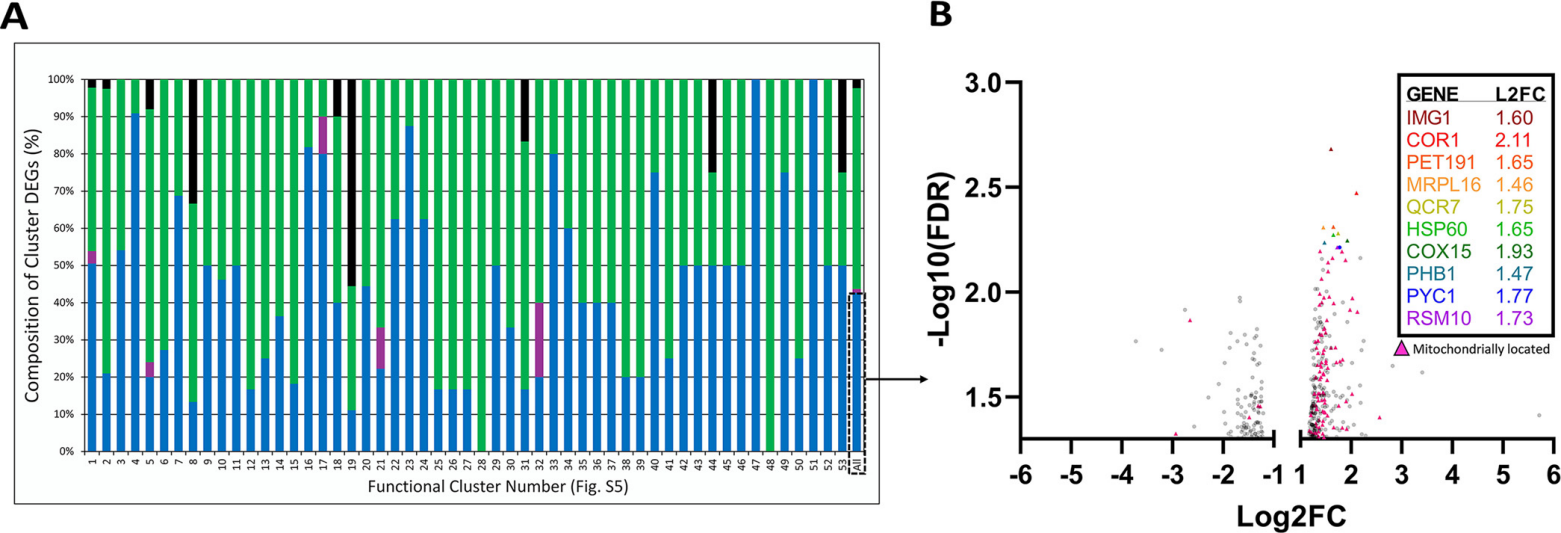
(A) Bar graph showing the gene categories within the differentially expressed gene list of S. cerevisiae (Sc) in response to consortium growth with both *T. delbrueckii* (Td) and *L. thermotolerans* (Lt). Percentages were calculated for the entire gene list (All), as well as for clusters 1 to 53. Black represents the percentage of genes common between all coculture conditions tested. Green represents the percentage of genes in common with the pairwise *T. delbrueckii* coculture. Purple represents the percentage of genes in common with the pairwise *L. thermotolerans* coculture. Blue represents the percentage of genes unique to consortium growth. (B) Volcano plot of differentially expressed genes in S. cerevisiae unique to consortium growth. Minimum −log_10_(FDR) of 1.3 and fold change cutoff of 1 and −1. Genes with the top 10 highest statistical significances are shown and color coded to the graph. Genes that are located in the mitochondria are represented by triangles.

**(iii) Growth in a consortium induces a combination of known pairwise responses as well as novel higher-order responses in S. cerevisiae.** To determine gene expression programs associated with higher-order interactions, we evaluated the DEGs of S. cerevisiae during growth within the three-species consortium. First, the extent to which the DEGs present during consortium growth could be predicted by the pairwise coculture DEGs was assessed by matching the genes in common between the pairwise and consortium conditions (Fig. 6A). Overall, 43% of the consortium DEGs were unique, i.e., expressed only during consortium growth and not under either pairwise condition, and 57% were present during pairwise coculture. Delving into this 57% of pairwise origin, 100% of the DEGs expressed during coculture with *L. thermotolerans* were expressed during consortium growth, and 73% of DEGs expressed during coculture with *T. delbrueckii* were expressed during consortium growth. This shows that pairwise ecological interactions are largely retained during consortium growth. The overall trend is that some prediction of interactions can be made from pairwise data; however, there indeed appears to be evidence for higher-order, or unpredictable, expression responses in S. cerevisiae.

Next, it was necessary to link the higher-order-associated DEGs with their broader cellular functions. The total list of DEGs was used to create a functional network, and the DEGs were labeled according to their commonality in the pairwise data sets (Fig. S5). This approach was followed to contextualize the higher-order DEGs within the broader functional network and more easily identify functional clusters that are uniquely associated with the higher-order response. To this end, each functional cluster was also represented by the percentage of DEGs that were in common with pairwise coculture or unique to the consortium setting (Fig. 6A and Fig. S5). It was found that many higher-order genes are functionally relevant to pairwise genes, illustrated by the distribution of higher-order and pairwise genes within their functional clusters (Fig. 6A and Fig. S5). For instance, cluster 9 consists of ergosterol biosynthesis genes, with half being present during the pairwise condition and the other half being stimulated by consortium growth. Other examples include clusters that are involved with autophagy (Fig. S5, cluster 11) and oxidative stress response (Fig. S5, cluster 18). This suggests that the cellular responses elicited during pairwise growth may be intensified in the consortium context.

10.1128/msphere.00436-22.6 FIG S5 Differentially expressed genes in S. cerevisiae in response to consortium culture with *L. thermotolerans* and *T. delbrueckii*. Nodes are colored according to log_2_ fold change values and sized according to statistical significance values. The borders of the nodes are colored according to which gene category they belong to, as described in Fig. 6. Borders are colored as follows: yellow, common to all conditions; purple, common to coculture with *L. thermotolerans*; green, common to coculture with *T. delbrueckii*; pink, unique to consortia. Image and network created in Cytoscape. Figure is for illustrative purposes only; please see interactive network files. Download FIG S5, PDF file, 0.7 MB. Copyright © 2022 Conacher et al. 2022 Conacher et al. https://creativecommons.org/licenses/by/4.0/ This content is distributed under the terms of the Creative Commons Attribution 4.0 International license.

Within this functional network there were also several clusters that consisted of majority higher-order interaction DEGs, which are notable as they may point to cellular responses that are indeed unique to higher-order interactions. This led us to perform functional enrichment and identify a network of the DEGs unique to the consortium growth condition in isolation (Fig. 6B and Fig. S6).

10.1128/msphere.00436-22.7 FIG S6 Differentially expressed genes in S. cerevisiae that are unique to consortium culture with *L. thermotolerans* and *T. delbrueckii*, with high statistical stringency (see Data Set S1). Nodes are colored according to log_2_ fold change values and sized according to statistical significance values. Figure is for illustrative purposes only; please see interactive network files. Download FIG S6, PDF file, 0.4 MB. Copyright © 2022 Conacher et al. 2022 Conacher et al. https://creativecommons.org/licenses/by/4.0/ This content is distributed under the terms of the Creative Commons Attribution 4.0 International license.

The data show a large proportion of these DEGs are localized to the mitochondria (38.4%) (Fig. 6B), with the most significant gene ontology process terms being mitochondrial translation and mitochondrial ATP synthesis-coupled electron transport (Data Set S1). Indeed, within the top five most statistically significant genes within this data set are two mitochondrial ribosomal large subunit genes (IMG1, MRPL16) and two mitochondrial respiratory chain complex III genes (QCR7, COR1) (Fig. 6B). As a whole, this suggests a metabolic shift to respiratory metabolism, with diversion of intracellular protein metabolism to energy generation strategies. The largest and most interconnected functional cluster, cluster 1 (Fig. S6), showed downregulation in cytoplasmic ribosomal genes and upregulation in mitochondrial ribosomal and translation genes. In accordance with this increase in mitochondrial translation machinery, there was also an increase in aminoacyl-tRNA ligases associated with mitochondrial translation (Fig. S6, cluster 10). In addition, cluster 2 (Fig. S6) showed upregulation of respiratory and ATP synthesis genes within the mitochondrion, and cluster 13 (Fig. S6) included upregulation of two major glucose-repressed transcriptional activators (HAP4 and HAP5) involved in regulation of respiratory metabolism. Cluster 16 (Fig. S6) displayed upregulation in mitochondrial organization-related genes as well as stress response genes. The opposing responses in mitochondrial and cytoplasmic translation machinery seen here are an interesting finding, as it is known that these processes are generally regulated in concert (44), and show a cellular priority for mitochondrial processes that generate energy. There are also signs of DNA replication stress and alterations to cell cycle checkpoints (Fig. S6, cluster 3), indicating impacts on proliferation rate as well. Further, protein trafficking within the cell was also upregulated, indicating impacts on protein turnover within the cell as well (Fig. S6, cluster 4).

Genes of the flocculin family, involved in cell adhesion and flocculation, were largely downregulated (Fig. S6, clusters 12 and 15). These genes have been highlighted in previous studies as a potential regulator of ecosystem dynamics and are an intuitive target as direct contact between cells influences the nature of their interactions (45–47). This seemingly indicates that S. cerevisiae may be avoiding direct cell contact with the other species within the culture, perhaps in an attempt to increase its chances to access limiting nutrients or to avoid cell wall-associated inhibition mechanisms of competing species (48).

In evaluating highly differentially expressed genes not necessarily associated with large clusters, the aromatic aminotransaminase, ARO9, was the most highly upregulated gene and is known to be induced by the presence of aromatic amino acids in growth media (49). The concentrations of aromatic amino acids (tyrosine, phenylalanine, and tryptophan) do not show significant differences between the monoculture and mixed cultures. This suggests a potential alternative function of this gene related to mixed-culture growth and has implications in terms of the generation of higher alcohols, which have previously been highlighted in other yeast-yeast interaction studies as potential signaling molecules and are of note since they are known to influence wine flavor and aroma (34, 35, 50, 51). The most downregulated genes included ZNF1, a glucose-repressed transcription factor, with regulatory roles in alternative carbon source utilization, respiration, and stress (52), as well as BDS1, a bacterially derived sulfatase responsible for utilization of sulfate esters, which suggests some impacts on sulfate import and metabolism (53).

Lastly, a cluster of interest related to the overarching aim of hypothesis generation is cluster 5, which consists of primarily uncharacterized open reading frames, which is significant given how well annotated the S. cerevisiae genome is. These would be good targets for annotation in a mixed-culture context, as opposed to the gold standard of high-throughput gene function characterization in monoculture.

## DISCUSSION

Higher-order interaction mechanisms within microbial ecosystems are poorly characterized. This study sought to contribute to our understanding of these mechanisms within a simplified wine yeast consortium. For this purpose, we characterized the emergence of higher-order interaction at the transcriptional level in a three-way yeast species interaction model.

S. cerevisiae differentially expressed a significant number of thiamine and NAD^+^ biosynthesis genes in response to coculture, regardless of the cohabitant type or number. Thiamine (vitamin B_1_) and its phosphorylated derivatives are important cofactors for enzymes involved in carbon metabolism, and thiamine is a growth factor of particular interest within fermentative processes because of its influence on glycolytic flux and fermentation efficiency (54–56). Competition for thiamine has also previously been highlighted in interactions of S. cerevisiae with *L. thermotolerans* and Hanseniaspora guilliermondii (7, 57). The pyruvate decarboxylase gene PDC5 was also upregulated within this list and is repressed by thiamine, signaling low intracellular thiamine levels (55, 58). Interestingly, the upregulation of *de novo* NAD^+^ biosynthesis and nicotinic acid uptake has direct links to thiamine biosynthesis, given that NAD^+^ is a necessary cofactor of thiamine biosynthesis enzymes and that these processes are regulated via the same protein, namely, Hst1p, an NAD^+^-dependent histone deacetylation protein (59). Thiamine accumulation has also been linked to providing protection against oxidative and osmotic stress, which is of relevance in the high-sugar growth medium used here (60). Further, NAD^+^ homeostasis plays a critical role in maintaining redox balance within the cell (61). The fact that these genes were impacted regardless of the species or number of cohabitant species alludes to these responses being indirectly linked to the presence of other species. It is likely that these responses were more a result of nutritional competition or other impacts of cohabitant species on the growth environment, as opposed to a direct and specific ecological interaction mechanism.

At the pairwise level, interesting differences in transcriptional responses of S. cerevisiae to either *L. thermotolerans* or *T. delbrueckii* were evident. There was a more extensive response to *T. delbrueckii* than there was to *L. thermotolerans*, with the majority of this response to *T. delbrueckii* being well aligned with known starvation responses, which is in agreement with previous studies (8, 9, 13). This may suggest that *T. delbrueckii* is more of a competitor for S. cerevisiae than *L. thermotolerans*, as already suggested by the relative growth rates. This transcriptional response is, however, displayed before the conditions reflect any imbalances in terms of nutrient availability and medium composition. The strong response of S. cerevisiae to *T. delbrueckii* has been noted before (8, 9, 13) and is hypothesized to be a result of very similar metabolism between the two species, given their close evolutionary history (62). *L. thermotolerans* has a similarly close phylogenetic relationship with S. cerevisiae and has been shown to have carbon and nitrogen preferences similar to those of S. cerevisiae and *T. delbrueckii* under the nutrient conditions applied here (33), although interstrain variability in nutritional requirements makes comparisons of this nature difficult (63). In the case of *T. delbrueckii*, the data point to a potential nutrient-sequestering response in S. cerevisiae, emulating a response to starvation, perhaps due to the similar metabolism of these two species, piquing a more robust response from S. cerevisiae. However, the precise mode of how this occurs would require further investigation. Considering the phenotypic evidence, the cell numbers of S. cerevisiae are largely identical between the two pairings, which shows that these differential responses resulted in successful maintenance of cell growth in the face of different competitors. The general similarity in the extracellular environment in terms of the metabolites tested here gives strong evidence to the hypothesis that the differential response to either species is a true biotic interaction impact.

Unique genes associated with the higher-order interaction context were observed. In the limited available literature, similar studies in bacteria have also found that pairwise population dynamics and metabolic cross-feeding data are correlated with what occurs within more complex systems but that there are indeed unpredictable nonlinear interactions that distort these interactions as well (16–20, 64). However, this has not been investigated at the transcriptomic level yet. Here, we highlight that there are unpredictable gene expression responses in S. cerevisiae within a yeast consortium at the early phases of growth. These responses were primarily associated with increasing mitochondrial translation and components of the electron transport chain needed for respiratory metabolism, as well as changes in cellular protein turnover. Alteration of S. cerevisiae’s metabolism to favor respiration is well studied in the context of cases where extracellular glucose drops below a particular level, although other nutrients may also play a role in this shift but are less well understood (65–67). However, the sugar concentrations show that glucose in the medium is nowhere near exhausted, and concentrations are highly similar between the compared conditions. Further, while nitrogen availability has also been implicated in stimulating respiratory metabolism (68), the available YAN was at similarly high concentrations between monoculture and mixed-culture samples. Interestingly, this switch to respiratory metabolism and adjusted protein turnover (particularly of the mitochondria) has been associated with longevity and aging mechanisms in S. cerevisiae and may perhaps be a mechanism to outlast its competitors (69). The trends suggest that S. cerevisiae employs a rapid diversification of its metabolism, as well as avoiding cell aggregation, in order to increase its chances of access to essential nutrients and thereby ensure its survival. In combination with the phenotypic and metabolite data presented here, the results add to a growing body of evidence that biotic stress is an extremely relevant selection pressure in the context of adaptive evolution.

### Conclusion.

Understanding yeast-yeast ecological interactions is a major research challenge, and the importance of characterizing and quantifying higher-order interactions in multispecies systems is clear. We know that S. cerevisiae strains of wine origin are competitive, dominate natural fermentations, and act as keystone drivers for the ecological dynamics of these systems. For the first time, we have shown the potential mechanisms behind how S. cerevisiae interacts within a multispecies yeast ecosystem at the transcriptional level. The functional networks generated by this study provide the most comprehensive functional overview of the complex mRNA transcriptome involved within these interactions. The data set provided here also contributes to a growing -omics database on yeast ecological interactions within mixed cultures. The limitations of the current study lie in the fact that only one (well-considered) time point was evaluated, and no confirmation of the relevance of the mRNA transcripts at the protein or metabolite level was done. Addressing these limitations should be prioritized in future studies while making use of the comprehensive transcriptional data reported here as a resource for hypothesis generation.

## MATERIALS AND METHODS

### Yeast strains.

Three yeast species representatives of wine-related origin were used to construct a synthetic yeast consortium. The three species were fluorescently labeled, each with a different fluorescent label, namely, S. cerevisiae VIN13 (Anchor Yeast, Cape Town, South Africa) labeled with TagRFP657, *L. thermotolerans* IWBT Y1240 (CBS: 16374) labeled with mTagBFP2, and *T. delbrueckii* LO544 (CRBO: LO544) labeled with enhanced green fluorescent protein (eGFP) (70). All yeast strains were stored as glycerol stocks (25% [wt/vol] glycerol) at −80°C. Prior to inoculation, glycerol stocks were streaked out onto Wallerstein Laboratory (WL) nutrient agar (Sigma-Aldrich, Johannesburg, South Africa) and incubated at 30°C for 3 days.

### Growth medium design.

The synthetic defined growth medium, yeast nitrogen base (YNB) with amino acids and ammonium sulfate (BD Difco, ThermoFisher Scientific), was adjusted to create a wine-like high-sugar cultivation medium that supported growth of all three yeast species within the consortium, referred to here as optimized YNB (OYNB). A summary of the growth medium design process is reported in the supplemental material (Table S1.1 and S1.2, Fig. S1, and Text S1). The optimized growth medium selected for culturing consisted of 6.7 g/L YNB with amino acids and ammonium sulfate, 100 g/L glucose, 100 g/L fructose, and 1× amino acid stock solution (Table S1.2 and Text S1).

10.1128/msphere.00436-22.2 FIG S1 Growth curves and viability decline comparisons during growth medium optimization. Download FIG S1, TIF file, 1.7 MB. Copyright © 2022 Conacher et al. 2022 Conacher et al. https://creativecommons.org/licenses/by/4.0/ This content is distributed under the terms of the Creative Commons Attribution 4.0 International license.

10.1128/msphere.00436-22.8 TABLE S1 (1) Summary of conditions tested for yeast nitrogen base growth medium design. (2) Amino acid stock solution composition for growth media used. Download Table S1, DOCX file, 0.01 MB. Copyright © 2022 Conacher et al. 2022 Conacher et al. https://creativecommons.org/licenses/by/4.0/ This content is distributed under the terms of the Creative Commons Attribution 4.0 International license.

10.1128/msphere.00436-22.1 TEXT S1 Description of the growth medium design method and results. Download Text S1, DOCX file, 0.02 MB. Copyright © 2022 Conacher et al. 2022 Conacher et al. https://creativecommons.org/licenses/by/4.0/ This content is distributed under the terms of the Creative Commons Attribution 4.0 International license.

### Preculture conditions.

Single colonies of each yeast strain were inoculated into 5 mL of yeast peptone dextrose (YPD) broth (Sigma-Aldrich, Johannesburg, South Africa) in a test tube and incubated on a test tube rotator at 30°C for 18 h. Four biological repeats were conducted, with a biological repeat defined as a culture originating from a separate colony. Cells were harvested by centrifugation, resuspended in OYNB, and transferred to 50 mL OYNB, at a concentration of 1 × 10^6^ cells · mL^−1^, in a 250-mL Erlenmeyer flask with a cotton plug and foil covering. The flask was incubated at 30°C, with agitation (150 rpm), for 8 h, until mid-exponential phase, after which the preculture was harvested by centrifugation at 5,000 × *g* for 5 min at room temperature and resuspended in OYNB at a volume of 10-fold less than the initial culture volume, before being inoculated.

### Culture conditions.

The culture conditions were selected to minimize the influence of abiotic stress to best evaluate biotic stress impacts. This was done by ensuring consistent extracellular conditions and biomass concentrations across different coculture combinations. This allowed for the evaluation of the response of S. cerevisiae to the presence of another species as opposed to nutrient limitation because of inconsistent concentrations of metabolizing yeast cells. Preculture biomass density was measured by optical density at 600 nm (OD_600_), and all cultures were inoculated to a final total density of an OD_600_ of 0.3. Four biological repeats of each species were inoculated into either single-, double-, or triple-species cultures (Table 1). Each species representative was inoculated at equal cell biomass, as determined by OD_600_ values. Cultures were conducted in 40 mL OYNB in 100-mL Erlenmeyer flasks with a cotton plug and foil covering. Growth medium was prewarmed to 30°C with agitation. Cultures were incubated at 30°C with agitation (150 rpm) until samples were removed for RNA extraction. All cultures were conducted on the same day to minimize batch variation.

**TABLE 1:** Summary of species composition and inoculation density of cultures tested

Culture	Inoculation density (OD_600_/species)	Species included
Single species	0.3	S. cerevisiae
Double species	0.15	S. cerevisiae plus *L. thermotolerans*
		S. cerevisiae plus *T. delbrueckii*
Triple species	0.1	S. cerevisiae plus *L. thermotolerans* plus *T. delbrueckii*

### Monitoring consortium population dynamics.

Consortium population dynamics were determined by quantitative flow cytometry as previously described (70), with the exception that all analyses were conducted on a single CytoFLEX flow cytometer (Beckman-Coulter), equipped with blue, violet, and red lasers. Briefly, viable cells, as determined by propidium iodide staining (Invitrogen, ThermoFisher, Waltham, MA, USA), were quantitatively measured by volumetric counting of fluorescently labeled yeast cells of each respective species.

### RNA Sequencing.

** (i) Sampling and RNA extraction.** The sampling point was chosen at a time where the total metabolic activities (taking sugar degradation as a proxy) between the monoculture and coculture settings were similar. To avoid intraspecific competition, the point was selected where all nutrients were in abundance (evidenced by metabolite data) but enough time for interaction had occurred. Samples were taken after approximately 7 h, when all cultures were in similar phases of early exponential growth, at a total cell concentration of 7.4 ± 0.1 log_10_ viable cells/mL (see Table S2.1 in the supplemental material), roughly a third of the way through the exponential phase. The sample point was selected to ensure that the monoculture would be in a growth phase comparable to those of both pairwise cultures as well as the consortium culture. For sampling, 2 mL of culture was removed, centrifuged at 5,000 × *g* for 3 min, resuspended in 500 μL cold RNAlater (ThermoFisher Scientific, South Africa), and stored at 4°C for 18 h until extraction. The sample supernatant was frozen at −20°C and kept for characterization of selected metabolites to contextualize the extracellular environment of the cells. Immediately before RNA extraction, a 1:1 volume of cold diethyl pyrocarbonate (DEPC)-treated phosphate-buffered saline (PBS) was added to the sample to reduce sample viscosity and aid in centrifugation of the samples. RNA extraction was performed using the Qiagen AllPrep DNA, RNA, and protein kit. The resultant RNA was checked for genomic DNA (gDNA) contamination by PCR of the ITS1/ITS4 region, with a positive gDNA control. RNA was stored at −80°C until sequencing.

**(ii) mRNA sequencing.** The total RNA samples were assessed for RNA integrity (RNA integrity number [RIN]) and quantity on the Bioanalyzer 2100 (Agilent Technologies, Waldbronn, Germany) using the RNA 6000 Nano Chip and reagents. mRNA was captured from 800 ng total RNA using the Dynabeads mRNA Direct Micro kit (ThermoFisher Scientific). The diluted mRNA was bound to the Dynabeads oligo(dT)_25_, washed, and eluted in 15 μL nuclease-free water. The Ion total transcriptome sequencing (RNA-Seq) kit v2 (ThermoFisher Scientific) was used to convert expressed mRNA transcripts into a representative cDNA library for strand-specific RNA sequencing on the Ion Torrent Ion S5 system. This library was purified and assessed for yield and fragment size distribution on the Agilent Bioanalyzer 2100 using the high-sensitivity DNA chip and kit (Agilent Technologies). The libraries were diluted to a target concentration of 80 pM and pooled in equimolar amounts for template preparation using the Ion 540 Chef kit (ThermoFisher Scientific). Enriched ion sphere particles were loaded onto an Ion 540 chip (ThermoFisher Scientific).

Massively parallel sequencing was performed on the Ion Torrent GeneStudio S5 Prime system using sequencing solution reagents according to the manufacturer’s protocol. Flow space calibration and basecaller analysis were performed using standard analysis parameters in the Torrent Suite version 5.12.2 software.

**(iii) Data analysis.** All sequencing data were processed and analyzed using Partek Flow software at the Central Analytical Facility for Next Generation Sequencing at Stellenbosch University. During preprocessing of the generated reads, two read-length cutoff parameter options, namely, 8 bp and 12 bp, were compared. It was found that the DEGs unique to each of the tested read-length cutoff conditions were mostly of borderline statistical significance under the cutoff condition under which they did not appear (see Data Set S1 in the supplemental material), with no notable differences in postalignment quality parameters. Therefore, instead of random selection of a particular cutoff parameter, the union of the gene sets produced by both of these cutoff conditions was used for functional analysis. This reduces introduction of bias in the analysis due to trimming (71). Labeled gene lists are provided in the supplemental material (Data Set S1) for separation of these gene sets, if required for other hypothesis testing.

Processed reads were mapped to a concatenated three-species genome, consisting of S. cerevisiae R64, *L. thermotolerans* CBS 6340, and *T. delbrueckii* CBS 1146, as it is planned to include analysis of all species in future, thereby keeping the analysis pipeline consistent. Read alignment was performed in two steps, first using STAR (2.6.1), followed by input of unaligned STAR reads into Bowtie2 (2.2.5), and finally combining the two alignment outputs. Non-uniquely mapped reads were randomly assigned to a particular portion of the reference. Aligned reads were then filtered to include only reads aligning to the S. cerevisiae genome. Reads mapping to annotated portions of the reference genome were then quantified by the expectation/maximization (E/M) algorithm applied in Partek. Quantified counts were then normalized by counts per million (CPM).

Gene set analysis (GSA) was performed to quantify differentially expressed genes, and the list was filtered to include genes with false-discovery rate (FDR) values of ≤0.05 and log_2_ fold change values of <−1 or >1. Monoculture samples were respectively compared to both pairwise samples, as well as to the tri-species (i.e., consortium) samples (Table 1).

### Extracellular metabolite analyses.

Sample supernatants were analyzed for glucose and fructose concentrations using enzymatic kits (Enzytec fluid d-fructose identifier [ID] no. E5120 [Roche, R-Biopharm]) and an automated analyzer (Konelab Arena 20XT; Thermo Electron Corporation, Finland) at the Chemical Analysis (CA) Laboratory of the Central Analytical Facility (CAF), Stellenbosch University. Glycerol and alcohols (methanol and ethanol), as well as selected organic acids (citric acid, tartaric acid, malic acid, succinic acid, lactic acid, and acetic acid), were quantified by high-performance liquid chromatography (HPLC) analysis (Agilent 1260 Infinity liquid chromatography system equipped with a μ-degasser [G1379B], 1260 binary pump [G1312B], 1260 standard autosampler [G1329B], 1260 thermostated column compartment [G1316A], 1260 diode array, and multiple wavelength detector [G4212C]) according to the method in reference 72 with chromatographic separation achieved using a Hi-Plex H (300- by 7.7-mm) column. Yeast assimilable nitrogen (YAN) was quantified using a Fourier transform near-infrared (FT-NIR) spectrometer equipped with a multipurpose analyzer (Bruker Optics, Ettlingen, Germany), where all controls and selections were made using the Opus/Quant software (Opus for Microsoft; Bruker Optics, Ettlingen, Germany). HPLC and YAN determinations were conducted at the CA Laboratory of CAF, Stellenbosch University. Amino acids (except cysteine and asparagine) were quantified at the Mass Spectrometry Unit of CAF, Stellenbosch University, using ultraperformance liquid chromatography (UPLC) (Waters Acquity) and photodiode array detection after derivatization with 6-aminoquinolyl-*N*-hydroxysuccinimidyl carbamate. Instrument control and data acquisition were performed by MassLynx software.

### Functional enrichment analysis and visualization of gene expression data.

For interpretation of the generated DEG lists, a cell-wide, pattern-based approach was taken, in order to better highlight major functional trends within the data set, rather than taking a specific, gene-for-gene approach which would focus on highly statistically significant gene targets in isolation. Functional enrichment analysis of the generated DEG lists was conducted through the STRING (v11) database functional enrichment tool. To generate a holistic view of the gene expression data, potential protein interaction networks were generated in STRING and interactive visualizations of the networks were created using Cytoscape (3.8.2) (73). The generated networks were visualized in Cytoscape (3.8.2) and clustered based on the distance matrix calculated from STRING global interaction scores, using the Markov CLustering (MCL) algorithm within the clusterMaker application (granularity = 2.5, unless otherwise stated). The clustered gene networks were colored by fold change values, while the size of the nodes was proportional to statistical significance. Functional enrichment analysis was then repeated on each cluster (with *n* > 4), and the results of this were summarized in a simple table (see Data Set S1 for overall and per-cluster functional enrichment analysis results). Using the clustered interaction networks, the main affected metabolic processes were identified, and highly statistically relevant genes could be contextualized within particular functional clusters (Fig. S2 to S6).

These networks were created for (i) DEGs that were commonly differentially expressed between both pairwise and consortium culture conditions, (ii) DEGs that were differentially expressed during pairwise coculture with *L. thermotolerans*, excluding DEGs in group i, (iii) DEGs that were uniquely differentially expressed during pairwise coculture with *T. delbrueckii*, excluding DEGs in group i, (iv) all DEGs that were differentially expressed during consortium growth, and (v) DEGs unique to consortium growth, filtered to remove DEGs that were present in pairwise DEG lists (Data Set S1).

### Data availability.

All supporting sequencing read data have been submitted to the National Center for Biotechnology Information (NCBI) Sequence Read Archive (SRA) under BioProject no. PRJNA783452. All generated network files which include all relevant metadata (gene lists, gene descriptions, gene-interaction significance values, FDR values, and specific fold change values) are available at the DOI link https://doi.org/10.25413/sun.20556033. Readers are encouraged to make use of these files to visualize the networks within Cytoscape as intended.
