# Supplementary material for: A Transcriptomic Analysis of Higher-Order Ecological Interactions in a Eukaryotic Model Microbial Ecosystem
Source: mSphere. 2022 Oct 19;7(6):e00436-22. doi: 10.1128/msphere.00436-22 (PMC9769528; doi:10.1128/msphere.00436-22)
Supplement: TABLE S2 [file msphere.00436-22-s0005.docx]

Table S2.1

| Culture | Biomass (Log10) | Citric acid (g/L) | Tartaric acid (g/L) | ANOVA* | Malic acid (g/L) | ANOVA * | Succinic acid (g/L) | Lactic acid (g/L) | Acetic acid (g/L) | Glucose (g/L) | ANOVA* | Fructose (g/L) | ANOVA* | Glycerol (g/L) | Methanol (g/L) | Ethanol (g/L) | ANOVA* | YAN  (mg N/L) | ANOVA* |
| --- | --- | --- | --- | --- | --- | --- | --- | --- | --- | --- | --- | --- | --- | --- | --- | --- | --- | --- | --- |
| Sc | 7.53 | ND | 0.048 | a | 3.12 | a | ND | ND | ND | 96.28 | a | 95.69 | a | ND | ND | 4.77 | a | 826.65 | a |
| ScLt | 7.42 | ND | 0.039 | a,  p = 0.67 | 3.17 | a,  p= 0.98 | ND | ND | ND | 97.38 | a,  p = 0.91 | 94.55 | a,  p = 0.87 | ND | ND | 3.92 | ab,  p = 0.24 | 804.13 | a,  p = 0.99 |
| ScLtTd | 7.36 | ND | 0.041 | a,  p = 0.83 | 3.11 | a,  p= 0.99 | ND | ND | ND | 98.86 | a,  p = 0.43 | 95.00 | a,  p = 0.97 | ND | ND | 3.38 | b,  p = 0.03 | 755.44 | a,  p = 0.95 |
| ScTd | 7.41 | ND | 0.041 | a,  p = 0.82 | 3.17 | a,  p=0.97 | ND | ND | ND | 97.73 | a,  p = 0.81 | 94.54 | a,  p = 0.87 | ND | ND | 3.86 | ab,  p= 0.19 | 775.14 | a,  p = 0.87 |

**Sc**: *Saccharomyces cerevisiae*; **Lt**: *Lachancea thermotolerans*; **Td**: *Torulaspora delbrueckii*

***:** The concentration of each metabolite was compared between cultures by ordinary one-way ANOVA, followed by Tukey's multiple comparisons test. The p values reported are for Sc (monoculture) compared to ScLt, ScTd, or ScLtTd (mixed culture) as calculated by Tukey's multiple comparison test.

Table S2.2

| **Culture** | **His** | **ANOVA-His** | **Arg** | **ANOVA-Arg** | **Ser** | **ANOVA-Ser** | **Gly** | **ANOVA-Gly** | **Asp** | **ANOVA-Asp** | **Glu** | **ANOVA-Glu** | **Thr** | **ANOVA-Thr** | **Ala** | **ANOVA-Ala** | **Pro** | **ANOVA-Pro** | **Lys** | **ANOVA-Lys** | **Tyr** | **ANOVA-Tyr** | **Met** | **ANOVA-Met** | **Val** | **ANOVA-Val** | **ILe** | **ANOVA-Ile** | **Leu** | **ANOVA-Leu** | **Phe** | **ANOVA-Phe** | **Gln** | **ANOVA-Gln** | **Tryp** | **ANOVA-Tryp** |
| --- | --- | --- | --- | --- | --- | --- | --- | --- | --- | --- | --- | --- | --- | --- | --- | --- | --- | --- | --- | --- | --- | --- | --- | --- | --- | --- | --- | --- | --- | --- | --- | --- | --- | --- | --- | --- |
| **ScLt** | 6.89 | a, p = 0.9998 | 449.87 | a, p = 0.1518 | 62.14 | b, p = 0.0258 | 25.01 | a, p = 0.8351 | 11.45 | b, p = 0.0036 | 61.62 | b, p = 0.0003 | 37.73 | b, p = 0.0125 | 121.60 | a, p = 0.9867 | 653.81 | a, p = 0.6782 | 31.94 | a, p = 0.6882 | ND | N/A | 17.33 | a, p = 0.9953 | 32.51 | ab, p = 0.7244 | 23.80 | b, p = 0.0141 | 22.99 | b, p = 0.0073 | 47.83 | a, p = 0.2026 | 181.09 | ab, p = 0.0733 | 113.29 | a, p = 0.8494 |
| **ScTd** | 13.95 | a, p = 0.2345 | 442.30 | a, p = 0.2536 | 60.70 | b, p = 0.0445 | 22.55 | a, p = 0.9998 | 11.83 | b, p = 0.0027 | 58.91 | b, p = 0.0006 | 44.30 | b, p = 0.0008 | 114.19 | a, p = 0.2 | 643.97 | a, p = 0.989 | 28.23 | a, p = >0.9999 | ND | N/A | 20.51 | a, p = 0.516 | 34.30 | ab, p = 0.1839 | 22.84 | b, p = 0.0368 | 22.94 | b, p = 0.0076 | 45.23 | a, p = 0.351 | 189.82 | b, p = 0.0284 | 99.64 | a, p = 0.9777 |
| **ScLtTd** | 7.66 | a, p = 0.9988 | 413.97 | a, p = 0.8939 | 62.31 | b, p = 0.0242 | 21.47 | a, p = 0.961 | 15.25 | b, p = 0.0002 | 68.19 | b, p = <0.0001 | 45.46 | b, p = 0.0005 | 115.90 | a, p = 0.3611 | 635.55 | a, p = 0.9786 | 26.03 | a, p = 0.8724 | ND | N/A | 15.98 | a, p = 0.992 | 36.56 | b, p =0.0162 | 24.71 | b, p = 0.0057 | 26.17 | b, p = 0.0007 | 44.02 | a, p = 0.4385 | 179.01 | ab, p = 0.0912 | 95.81 | a, p = 0.9916 |
| **Sc** | 7.17 | a | 398.21 | a | 46.57 | a | 22.74 | a | 2.09 | a | 27.84 | a | 22.89 | a | 122.99 | a | 640.26 | a | 28.42 | a | ND | N/A | 16.72 | a | 30.86 | a | 17.22 | a | 13.83 | a | 33.47 | a | 137.29 | a | 86.35 | a |

**Sc**: *Saccharomyces cerevisiae*; **Lt**: *Lachancea thermotolerans*; **Td**: *Torulaspora delbrueckii*

***:** The concentration of each amino acid was compared between cultures by ordinary one-way ANOVA, followed by Tukey's multiple comparisons test. The p values reported are for Sc (monoculture) compared to ScLt, ScTd, or ScLtTd (mixed culture) as calculated by Tukey's multiple comparison test.
