# Supplementary figures and images for: A Transcriptomic Analysis of Higher-Order Ecological Interactions in a Eukaryotic Model Microbial Ecosystem
Source: mSphere. 2022 Oct 19;7(6):e00436-22. doi: 10.1128/msphere.00436-22 (PMC9769528; doi:10.1128/msphere.00436-22)

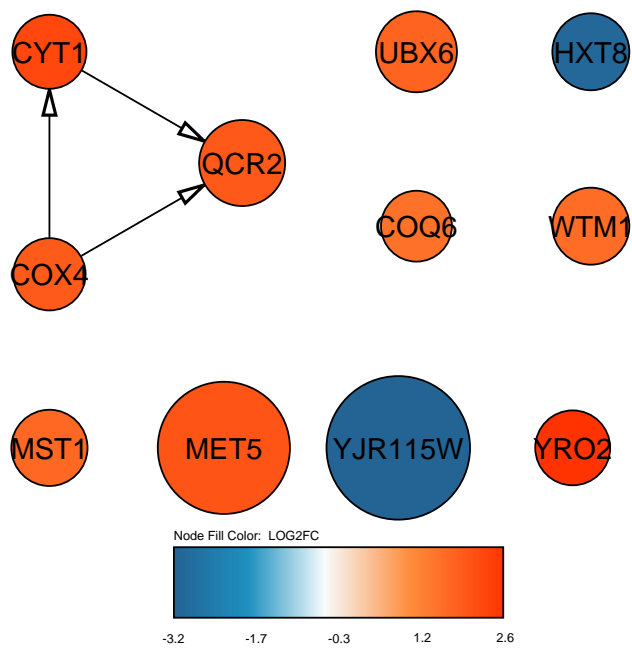

Supplement: FIG S3 [file msphere.00436-22-s0007.pdf]

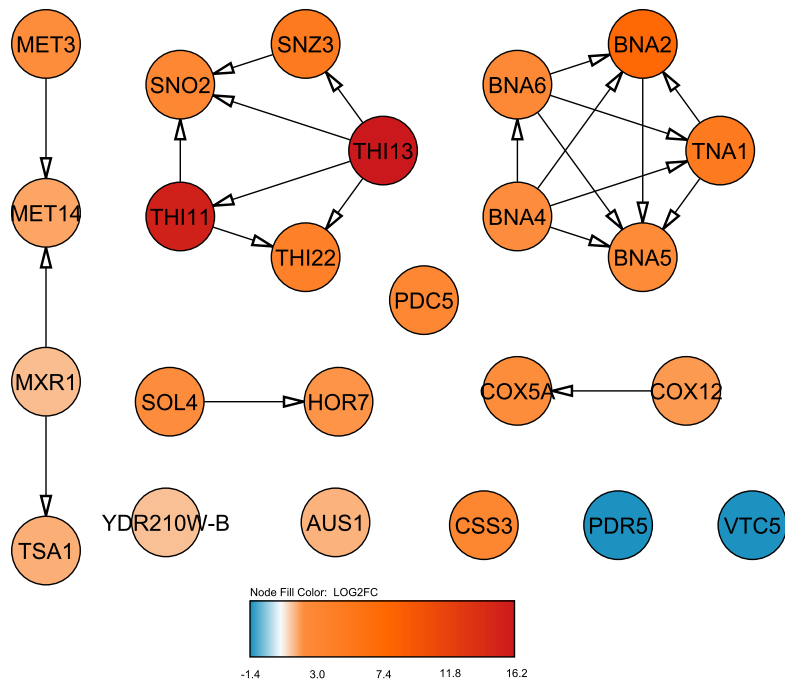

Supplement: FIG S2 [file msphere.00436-22-s0006.pdf]

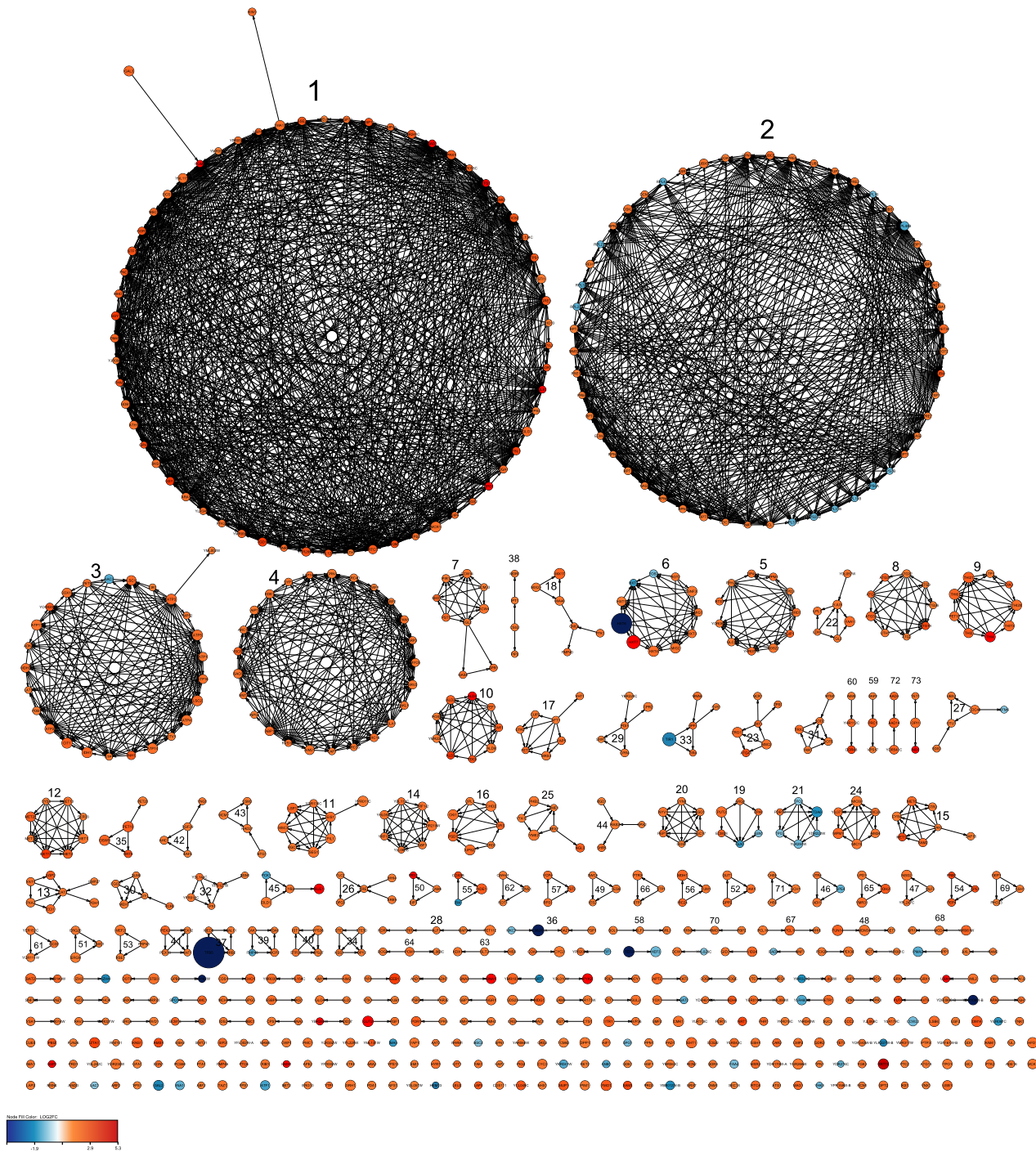

Supplement: FIG S4 [file msphere.00436-22-s0008.pdf]

2

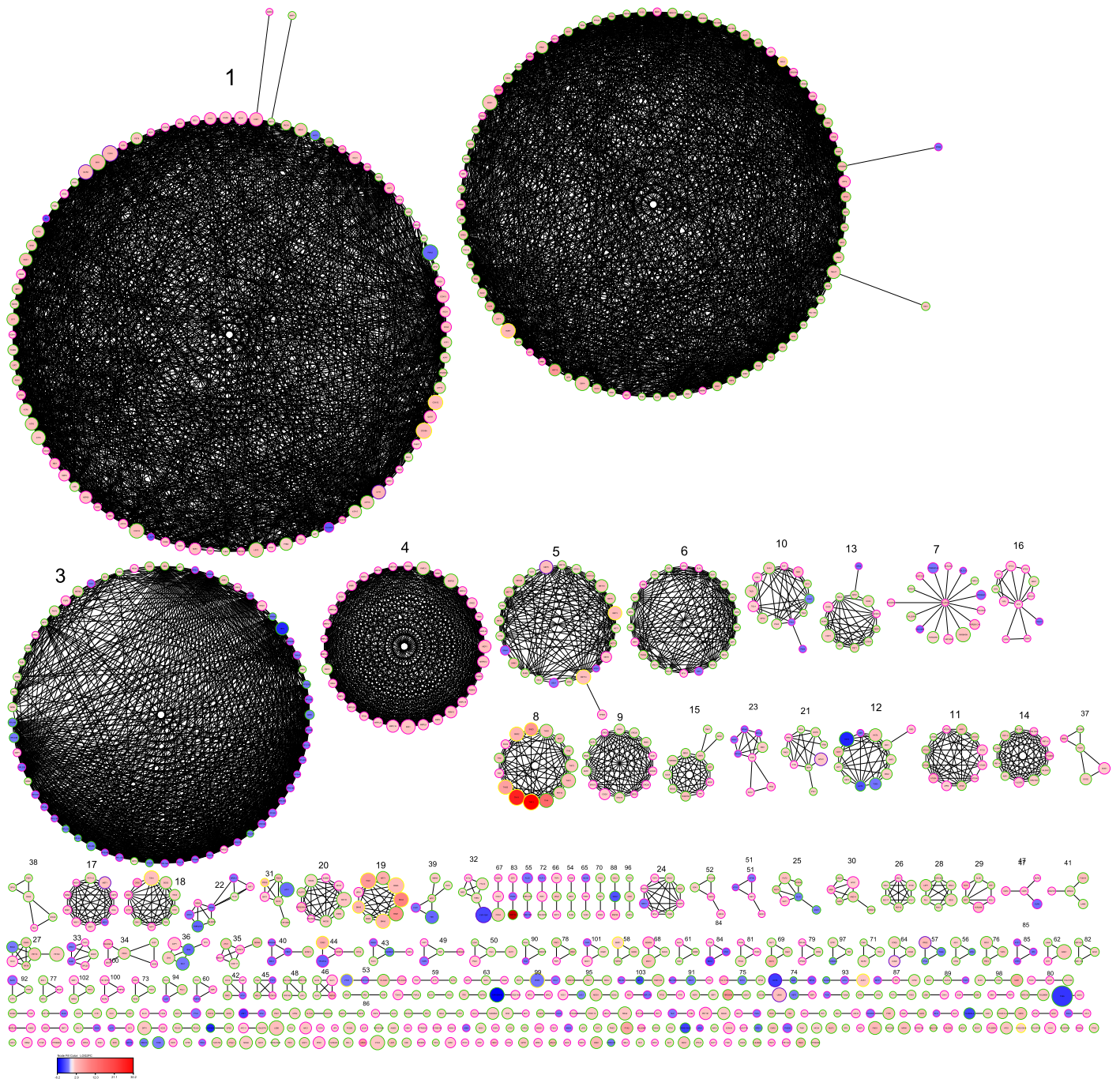

Supplement: FIG S5 [file msphere.00436-22-s0009.pdf]

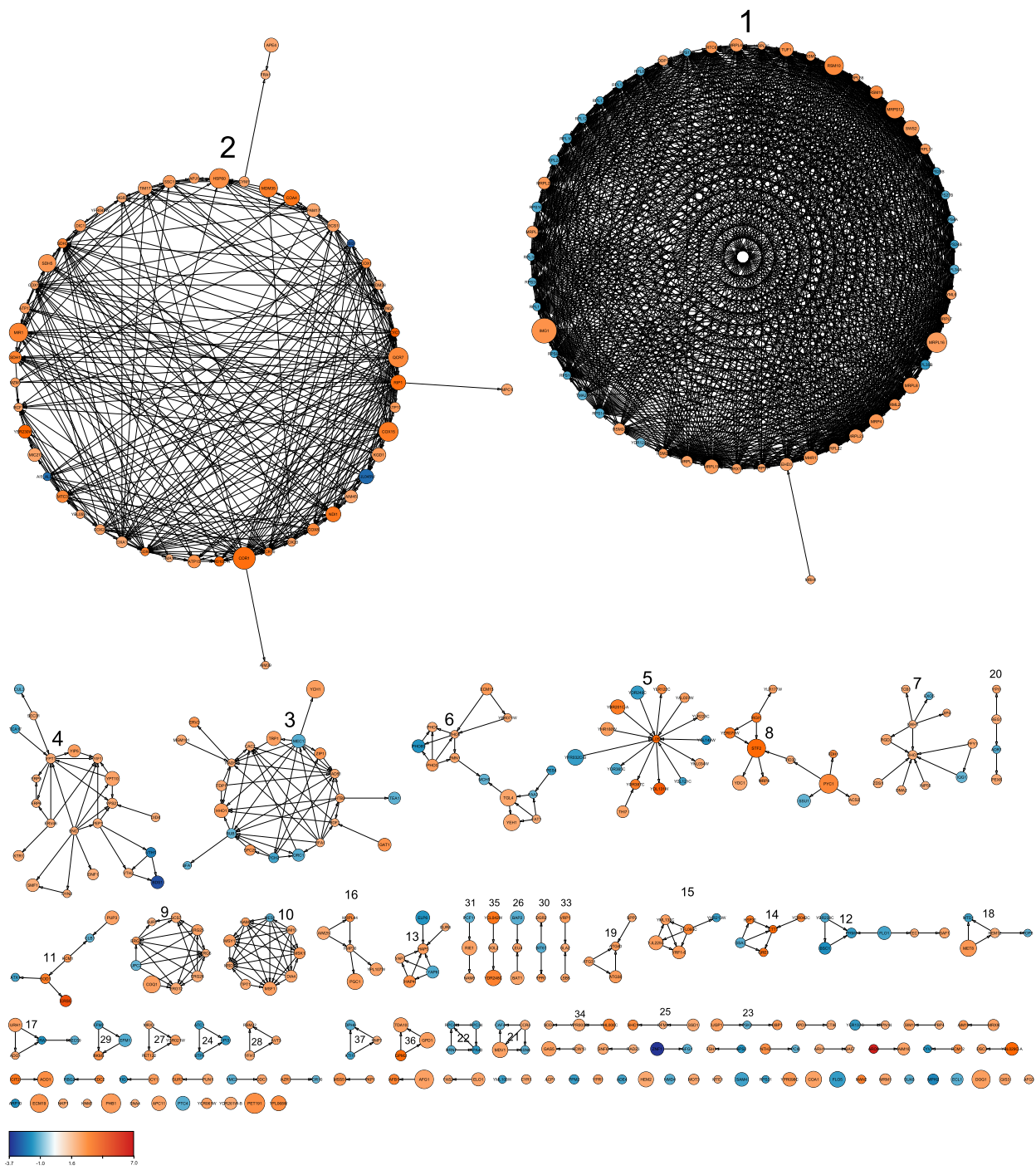

Supplement: FIG S6 [file msphere.00436-22-s0010.pdf]

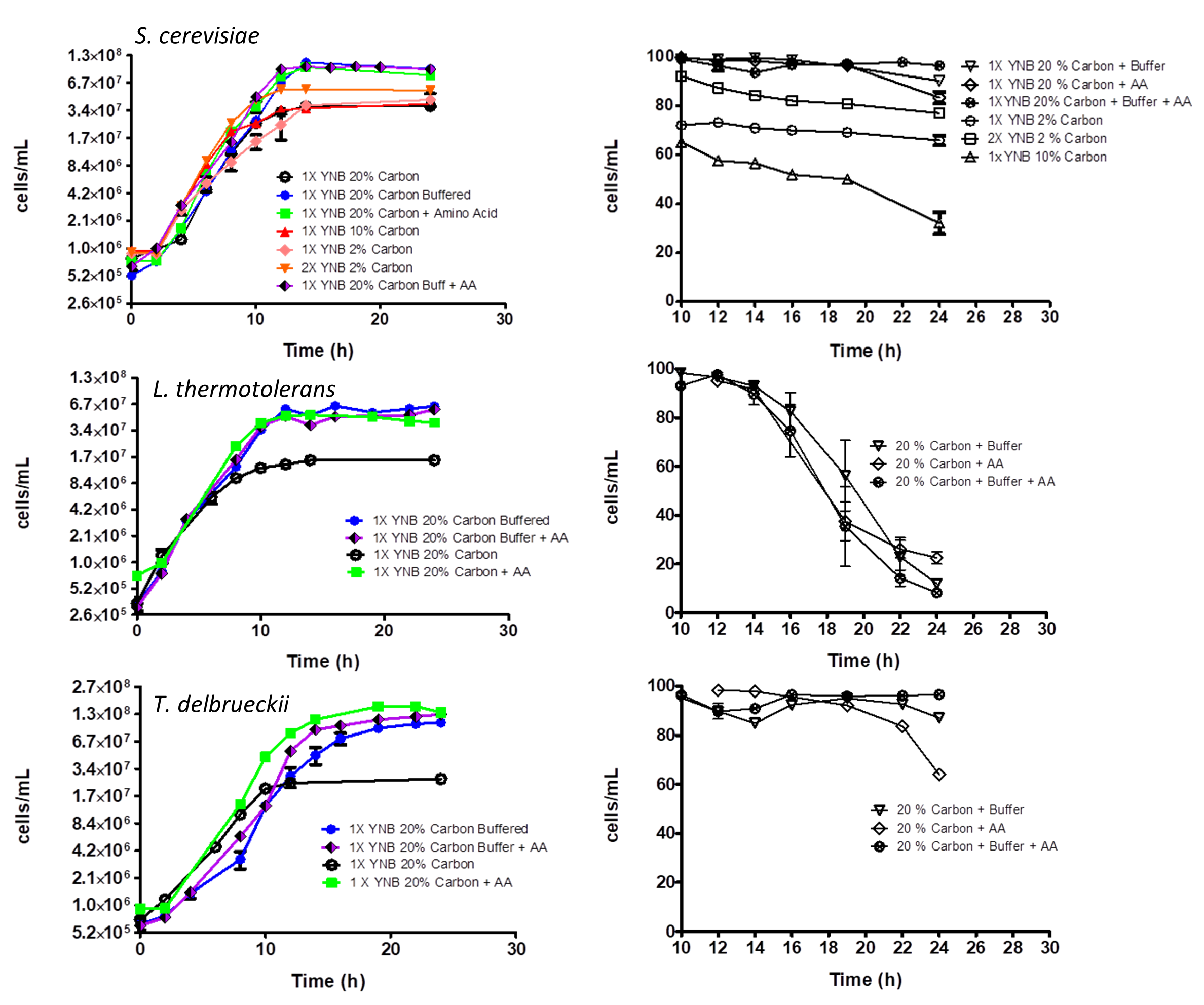

Supplement: FIG S1 [file msphere.00436-22-s0004.tif]
