# Supplementary material for: A Transcriptomic Analysis of Higher-Order Ecological Interactions in a Eukaryotic Model Microbial Ecosystem
Source: mSphere. 2022 Oct 19;7(6):e00436-22. doi: 10.1128/msphere.00436-22 (PMC9769528; doi:10.1128/msphere.00436-22)
Supplement: TABLE S1 [file msphere.00436-22-s0003.docx]

Table S1.1

| Condition | Carbon Source | YNB Concentration | Additional Components | Species Tested |
| --- | --- | --- | --- | --- |
| Carbon Source Concentration | Low: 2 % (w/v) | 1X: 6.7 g/L | None | *S. cerevisiae* |
|  | Medium: 10 % (w/v) | 1X: 6.7 g/L | None | *S. cerevisiae* |
|  | High: 20 % (w/v) | 1X: 6.7 g/L | None | *S. cerevisiae,*  *L. thermotolerans,*  *T. delbrueckii* |
| YNB Concentration | Low: 2 % (w/v) | 1X: 6.7g/L | None | *S. cerevisiae* |
|  |  | 2X: 13.4 g/L | None | *S. cerevisiae* |
| Buffering | High: 20 % (w/v) | 1X: 6.7g/L | 100mM Potassium phosphate buffer | *S. cerevisiae,*  *L. thermotolerans,*  *T. delbrueckii* |
| Additional amino acids** | High: 20 % (w/v) | 1X: 6.7g/L | Amino acid stock solution | *S. cerevisiae,*  *L. thermotolerans,*  *T. delbrueckii* |
| Buffering + Additional amino acids | High: 20 % (w/v) | 1X: 6.7g/L | 100mM Potassium phosphate buffer + Amino acid stock solution | *S. cerevisiae,*  *L. thermotolerans,*  *T. delbrueckii* |

**: Optimized YNB used in cultures

Table S1.2

| Stock of amino acids (150X Stock) | |
| --- | --- |
| Prepare in 1l of NaHCO3 2% (20g/l) and add amino acids slowly in following order | |
|  | **[g/l]** |
| tyrosine | 1.40 |
| tryptophane | 13.70 |
| isoleucine | 2.50 |
| aspartic acid | 3.40 |
| glutamic acid | 9.20 |
| arginine | 28.60 |
| leucine | 3.70 |
| threonine | 5.80 |
| glycine | 1.40 |
| glutamine | 38.60 |
| alanine | 11.10 |
| valine | 3.40 |
| methionine | 2.40 |
| phenylalanine | 2.90 |
| serine | 6.00 |
| histidine | 2.50 |
| lysine | 1.30 |
| cysteine | 1.00 |
| proline | 46.80 |
