# Supplementary material for: A Transcriptomic Analysis of Higher-Order Ecological Interactions in a Eukaryotic Model Microbial Ecosystem
Source: mSphere. 2022 Oct 19;7(6):e00436-22. doi: 10.1128/msphere.00436-22 (PMC9769528; doi:10.1128/msphere.00436-22)
Supplement: TEXT S1 [file msphere.00436-22-s0002.docx]

# Supplementary Materials for: A transcriptomic analysis of higher-order ecological interactions in a eukaryotic model microbial ecosystem

# Links to supplementary files

**Supplementary File 1 and Cytoscape network files: DOI:** 10.25413/sun.20556033

**S1 Growth medium design**

A range of conditions were first tested in *S. cerevisiae*, after which selected conditions were tested in *L. thermotolerans* and *T. delbrueckii* (Table S1.1). Similar to Synthetic Grape Must (SGM), the carbon source was comprised of equal concentrations of glucose and fructose. The impact of carbon source concentration, YNB concentration, the addition of amino acids (Table S1.2), and buffering at pH 6 were evaluated in *S. cerevisiae*. A low [2 % (w/v)], medium [10 % (w/v)], and high [20 % (w/v)] carbon source concentration was tested. The concentration of YNB was increased 2-fold in the 2 % carbon medium to adjust carbon:nitrogen ratio. Next, in the high carbon source medium, buffering at pH 6, with 100 mM Potassium Phosphate buffer was evaluated. The addition of complete amino acid stock, based on SGM, was tested. Finally, the effect of combining buffering and additional amino acids was determined.

**S1 Growth curves and viability decline comparisons during growth media optimization (Fig. S1)**

Initial growth experiments in high sugar YNB (with amino acids, 0.5 % ammonium sulphate, 20 % carbon source) resulted in low biomass yields across all three tested species, and mixed species cultures showed similar attenuated growth (Fig. S1). To attempt to improve the biomass yields and assess viability, a number of settings were first screened in *S. cerevisiae*. Initially, lowering the sugar concentration to 2 % YNB resulted in similar low biomass yields and loss in viability from the 10 hour point in *S. cerevisiae*. Increasing the carbon concentration to 10 % did not improve biomass and caused rapid decline in viability in *S. cerevisiae*. It was hypothesized that the carbon:nitrogen ratio was not balanced, and rapid decline in pH may be causing the suboptimal growth. In the low sugar YNB, the concentration of YNB was doubled, and this resulted in better biomass yields and improved viability. This indicated that a limiting nutrient in the YNB components may be attenuating growth. Applying this to improve the high sugar medium, additional amino acids in the form of a complete amino acid stock, usually used in SGM growth medium, were added. This improved biomass yield and allowed *S. cerevisiae* cells to remain viable until at least 20 hours of growth. Secondly, the role of pH was investigated by buffering the high sugar YNB medium to pH 6. This showed similar improvements in biomass yields and viability. The addition of both buffer and amino acids to high sugar YNB did not improve the biomass yields and viability over addition of only buffer or amino acids. The three best performing high sugar YNB variants, namely, addition of amino acids, or buffer, or both amino acids and buffer were further evaluated in *L. thermotolerans* and *T. delbrueckii*. There were similar improvements in biomass yields and all tested variations showed similar viability. It was however observed that *T. delbrueckii* flocculated severely when grown in the presence of the buffer. This has negative implications for the use of flow cytometry quantitation. Therefore, since the addition of amino acids alone appeared to create an appropriate growth medium for all three species, it was decided to move forward with this growth medium.
